# Oxidative stress induces transient O‐GlcNAc elevation and tau dephosphorylation in SH‐SY5Y cells

**DOI:** 10.1111/jcmm.12910

**Published:** 2016-07-26

**Authors:** Emese Kátai, József Pál, Viktor Soma Poór, Rupeena Purewal, Attila Miseta, Tamás Nagy

**Affiliations:** ^1^ Department of Laboratory MedicineUniversity of PécsPécsHungary; ^2^ Department of NeurosurgeryUniversity of PécsPécsHungary; ^3^ Neuronal Networks GroupCollege of Medical and Dental SciencesUniversity of BirminghamBirminghamUK; ^4^ Department of Forensic MedicineUniversity of PécsPécsHungary; ^5^ Department of PediatricsWest Virginia UniversityMorgantownWVUSA; ^6^ János Szentágothai Research CentreUniversity of PécsPécsHungary

**Keywords:** O‐GlcNAc, oxidative stress, stress response, Alzheimer's disease, tau phosphorylation

## Abstract

O‐linked β‐*N*‐acetlyglucosamine or O‐GlcNAc modification is a dynamic post‐translational modification occurring on the Ser/Thr residues of many intracellular proteins. The chronic imbalance between phosphorylation and O‐GlcNAc on tau protein is considered as one of the main hallmarks of Alzheimer's disease. In recent years, many studies also showed that O‐GlcNAc levels can elevate upon acute stress and suggested that this might facilitate cell survival. However, many consider chronic stress, including oxidative damage as a major risk factor in the development of the disease. In this study, using the neuronal cell line SH‐SY5Y we investigated the dynamic nature of O‐GlcNAc after treatment with 0.5 mM H_2_O_2_ for 30 min. to induce oxidative stress. We found that overall O‐GlcNAc quickly increased and reached peak level at around 2 hrs post‐stress, then returned to baseline levels after about 24 hrs. Interestingly, we also found that tau protein phosphorylation at site S262 showed parallel, whereas at S199 and PHF1 sites showed inverse dynamic to O‐Glycosylation. In conclusion, our results show that temporary elevation in O‐GlcNAc modification after H_2_O_2_‐induced oxidative stress is detectable in cells of neuronal origin. Furthermore, oxidative stress changes the dynamic balance between O‐GlcNAc and phosphorylation on tau proteins.

## Introduction

Cells maintain their intracellular milieu within a quite narrow range. This is a dynamic equilibrium which stabilizes the cells’ internal environment in response to the alterations of external conditions. Cells exposed to any damage react with a complex mechanism called stress response which varies by the actual tolerance level of the given cell type and the duration and severity of the stress 1. When the impairment of the cells does not cause immediate cell death (necrosis), the cells may recover and adapt to the changed environment 2. When complete adaptation is not possible, stress response will include hypertrophy, hyperplasia, atrophy and metaplasia or activation of death signalling pathways: apoptosis, necrosis or autophagy cell death 1, 3, 4. Not surprisingly, dysfunctions of the adaptive mechanisms play an important role in many diseases such as ischaemia, diabetes, atherosclerosis, neurodegenerative disorders and tumours.

Stress response induces and regulates a variety of intracellular adaptive mechanisms ranging from receptor binding, signal transduction, gene transcription to protein synthesis 5. These mechanisms contribute to the survivability of the cells by controlling cell cycle, repairing/stabilizing functional proteins, removing of damaged molecules, mobilizing stored resources and modifying metabolic pathways 3, 6, 7. Cellular proteins are regulated in these processes at multiple levels; altered protein degradation (*e.g*. activation of caspases) or synthesis (transcriptional changes, unfolded protein response) and functional changes 8, 9, 10. Post‐translational modifications such as phosphorylation, glycosylation and acetylation also play a critical role in these processes, as they can reversibly or irreversibly modify the structure, the stability and the function of proteins 11.

Recently, several studies indicated the involvement of a special post‐translational modification in stress response: O‐linked β‐*N*‐acetylglucosamine or O‐GlcNAc modification 12, 13. Upon O‐GlcNAc modification, a single *N*‐acetylglucosamine is attached to the hydroxyl group of serine or threonine moieties of cytosolic and nuclear proteins 14. This modification is a dynamic and reversible process and was shown to impact so far more than 1000 cytoplasmic, nuclear and mitochondrial proteins such as NF‐Κb, PI3‐kinase, endothelial nitric oxide synthase, O‐GlcNAc transferase (OGT), α‐tubulin, c‐myc and heat shock protein 70 15, 16. It plays a critical role in the regulation of signal transduction, transcriptional activity, cell‐cycle regulation, protein degradation and glucose metabolism 15. Previous studies have shown that increased O‐GlcNAc has a protective role in acute stress, best characterized in cardiac cells 17. Recent findings suggest that O‐GlcNAc is also abundant in neuronal tissues 18 and it was shown that chronic imbalance between O‐GlcNAc and phosphorylation on tau proteins is a major element in the pathophysiology of Alzheimer's disease 19. Although it was shown that O‐GlcNAc is dynamic and is induced by excitatory neuronal stimuli 18, the regulation of O‐GlcNAc during acute stress in neuronal cells is far from completely understood.

The aim of this study was to investigate the dynamic change in O‐GlcNAc in the neuronal cell line SH‐SY5Y after H_2_O_2_‐induced oxidative stress. We found that intracellular O‐GlcNAc rapidly elevated after treatment with 0.5 mM H_2_O_2_ for 30 min., reached peak level then declined close to the basal level. Moreover, reciprocal relationship between O‐GlcNAc and phosphorylation of tau protein after oxidative stress was directly demonstrated. These findings suggest that the pathophysiology of neurodegenerative diseases might be connected to cellular stress adaptation mechanisms.

## Materials and methods

### Cell line and culture conditions

SH‐SY5Y neuroblastoma cells (ATCC CRL‐2266 human neuroblastoma) were grown in a 1:1 mixture of EMEM and Ham's F12 medium supplemented with 10% foetal bovine serum (FBS), 1% non‐essential amino acids, penicillin (100 U/ml) and streptomycin (100 μg/ml). The cells were incubated at 37°C, in 95% air–5% CO_2_ atmosphere in a humidified incubator. Subculturing was performed in every 2–3 days and fresh medium was replaced 12–24 hrs prior to each experiment. The experiments were performed on the cells at 80% or less confluency. Oxidative conditions were chosen based on experimental set‐ups found in the literature 20, 21 and our pilot experiments (data not shown). Our aim was to decrease the viability to ~75% by the treatment. Thus, cells were treated with 0.5 mM H_2_O_2_ for 30 min. in serum‐free medium to avoid rapid H_2_O_2_ degradation by antioxidants present in FBS. After the treatment, the medium was replaced by complete medium and the cells were incubated as described above for the following recovery times: 0, 30, 60 min. and 2, 4, 24, 48 and 72 hrs.

### Western blot analysis

The treated cells were washed twice in ice‐cold PBS buffer and harvested in RIPA buffer [10 mM Tris pH 7.2, 100 mM NaCl, 1 mM ethylenediaminetetraacetic acid (EDTA), 1 mM ethylene glycol‐bis(2‐aminoethylether)‐N,N,N',N'‐tetraacetic acid (EGTA), 0.1% SDS, 1% Triton‐X 100, 0.5% deoxycholate, 10% glycerol, protease inhibitor cocktail: 1 tablet/10 ml (Roche Applied Science, Penzberg, Germany)], kept on ice for 30 min. and centrifuged for 10 min. at 4°C at 14,000 × g. From the supernatant the total protein concentration was determined using Bio‐Rad *Dc* Assay Kit. Proteins were separated on 7.5% SDS‐PAGE and transferred onto polyvinylidene difluoride membranes (Millipore, Billerica, MA, USA). The same amount of protein of each sample was confirmed by SYPRO Ruby Protein Blot Stain (Bio‐Rad, Hercules, CA, USA) on the membranes. Blots were probed with the anti‐O‐GlcNAc antibody CTD110.6 (monoclonal mouse IgM, 1:2000; Sigma‐Aldrich, St. Louis, MO, USA) in 1% casein blocking buffer and followed with HRP conjugated goat antimouse IgM (1:5000; Thermo Fisher Scientific, Waltham, MA, USA). Blots were also probed with mouse (monoclonal) anti‐tau antibody (1:250) and rabbit (polyclonal) anti‐tau [Ps^199^] phophospecific antibody (1:1000; Thermo Fisher Scientific), rabbit (polyclonal) anti‐tau [Ps^262^] antibody (1:1000; Abcam, Cambridge, MA, USA) and PHF1 (anti‐tau Ps^396/404^) (1:200; gift from Dr. P. Davies, Albert Einstein College of Medicine, Bronx, NY, USA) according to the manufacturer's protocol. The blots were developed using Femto chemiluminescent substrate (Thermo Fisher Scientific) and the signal was visualized by Kodak Image Station 2000R. Kodak 1D and ImageJ analysis software were used to quantify the intensity of bands.

### Cell viability test

Cells were lifted by trypsin and quickly washed 2× in ice‐cold PBS. Approximately 10^6^ cells/sample were stained with Propidium Iodide and Annexin V‐FITC according to the manufacturer's instruction (BD Pharmingen, Heidelberg, Germany). The fluorescence signal of Propidium‐Iodide dye was detected at FL3 channel (620 nm), and FITC Annexin V intensity was detected at FL1 channel (525 nm) with a Cytomic FC 500 flow cytometer (Beckman Coulter, Fullerton, CA, USA). Defining the quadrants of dead cells (positive for propidium iodide) and live cells (minimal staining for both Propidium Iodide and FITC Annexin V) was performed on control samples and identical boundaries were utilized for all samples.

### Intracellular ROS detection

Intracellular reactive oxygen species (ROS) levels were measured by fluorescence using CM‐H2DCFDA (Thermo Fisher Scientific) fluorescence dye as a ROS probe. SH‐SY5Y cells were cultured as mentioned above. Before the experiments, the cells were lifted from the flasks by 0.25% trypsin/0.5 mM EDTA/PBS then immediately washed with pre‐warmed complete media to neutralize trypsin. Next, the cells were quickly washed in Hank's balanced salt solution (HBSS), and resuspended in HBSS containing 1% bovine serum albumin, 1.2 mM Ca^2+^, 1.0 mM Mg^2+^ and 2 μM CM‐H2DCFDA. Samples were incubated in the dark at room temperature for 30 min. Cell suspensions were gently mixed every 10 min. during the incubation to prevent the attachment to the vessels’ wall. Following two wash steps to remove the excess of the fluorescence dye, the cells were resuspended in HBSS. Fluorescence signal was measured at 25°C by F4500 fluorescence spectrophotometer (Hitachi High‐Technologies Europe, Krefeld, Germany) at excitation wavelength of 490 nm and emission wavelength of 525 nm. Baseline level of fluorescence was recorded for ~1 min. before the addition of 0.5 mM H_2_O_2_ or vehicle. The relative level of ROS was expressed as F/F_0_ values. Fluorescence signal was monitored for 10 min. following peroxide or vehicle treatment.

### Real‐time RT‐PCR analysis

Total RNA was isolated from SH‐SY5Y neuroblastoma cells using RNeasy Mini Kit (Qiagen, Hilden, Germany). RNA quantity was determined using a Nanodrop Implen Nanophotometer. Reverse transcription into cDNA was performed with iScript cDNA Synthesis Kit (Bio‐Rad). Pre‐designed TaqMan assay (Roche Applied Science) was used to determine mRNA expression levels of human OGT and glucosamine‐fructose‐6‐phosphate aminotransferase (GFAT). As a reference gene, human porphobilinogen deaminase (PBGD) was used. Amplification primers and hydrolysis probes were designed using ProbeFinder software (www.universalprobelibrary.com). Sequences of the forward and reverse primers of OGT were 5′‐AGACGATGGCACAAACTTCC‐3′ and 5′‐ATCAGCTGCTTTTCCATTGC‐3′, the hydrolysis probe was UPL #29. Sequences of the forward and reverse primers of GFAT were 5′‐TGAGATTGGTGTGGCCAGTA‐3′ and 5′‐GGCAAACATCACAAGGGATAC‐3′, the hydrolysis probe was U PL #20. The sequences of the reference gene PBGD were as follows: sense 5′‐TGCCAGAGAAGAGTGTGGTG‐3′, antisense 5′‐AGCCGGGTGTTGAGGTTT‐3′, the hydrolysis probe was UPL #24. Real‐time PCR was performed in a LightCycler^®^ thermal cycler (Roche Applied Science). Each reaction was performed in a 20 μl volume, using the LightCycler Taqman Master Mix (Roche Applied Science). Both target and reference genes were amplified with efficiencies near 100% and within 5% of each other. For the relative gene expression analysis, the 2ΔΔCt (Livak) method was used. The expression level of the gene of interest was compared with the level of PBGD in each sample.

### Immunofluorescence microscopy

Cells were grown on coverslips and after oxidative stress/recovery treatments they were washed twice in ice‐cold PBS. Next, the cells were fixed in 10% PBS‐buffered formaldehyde for 30 min. at room temperature, subsequently washed with PBS. To avoid formaldehyde autofluorescence, the coverslips were quenched with 50 mM ammonium chloride for 10 min. Cells were permeabilized with 0.1% Triton‐X 100 for 5 min. Non‐specific sites were blocked with 5% bovine serum albumin (Sigma‐Aldrich) in PBS for 5 min. and then the coverslips were incubated at room temperature with CTD110.6 monoclonal antibody for 30 min. at a dilution of 1:100 in 5% BSA/PBS. After rinsing three times with PBS, the samples were incubated with Alexa Fluor 594 goat antimouse IgM secondary antibody (1:200; Thermo Fisher Scientific) for 30 min. in dark. Nuclei were counterstained with Hoechst dye at a dilution of 1:5000 for 15 min. at room temperature. Finally, coverslips were mounted with Vectashield (Vector Laboratories, Burlingame, CA, USA) mounting medium. Image acquisition was performed with a Zeiss Axiovert 35 (Carl Zeiss Microscopy GmbH, Jena, Germany) inverted fluorescent microscope with CellD (Olympus, Tokyo, Japan) software.

### Data analysis

Data are presented as means ± S.E.M. throughout. Comparisons were performed with repeated measures one‐way anova and statistically significant differences between groups were defined as *P* < 0.05 and are indicated in the legends of figures.

## Results

### Effect of treatment with 0.5 mM H_2_O_2_ for 30 min. on cell survival

**Figure 1:**
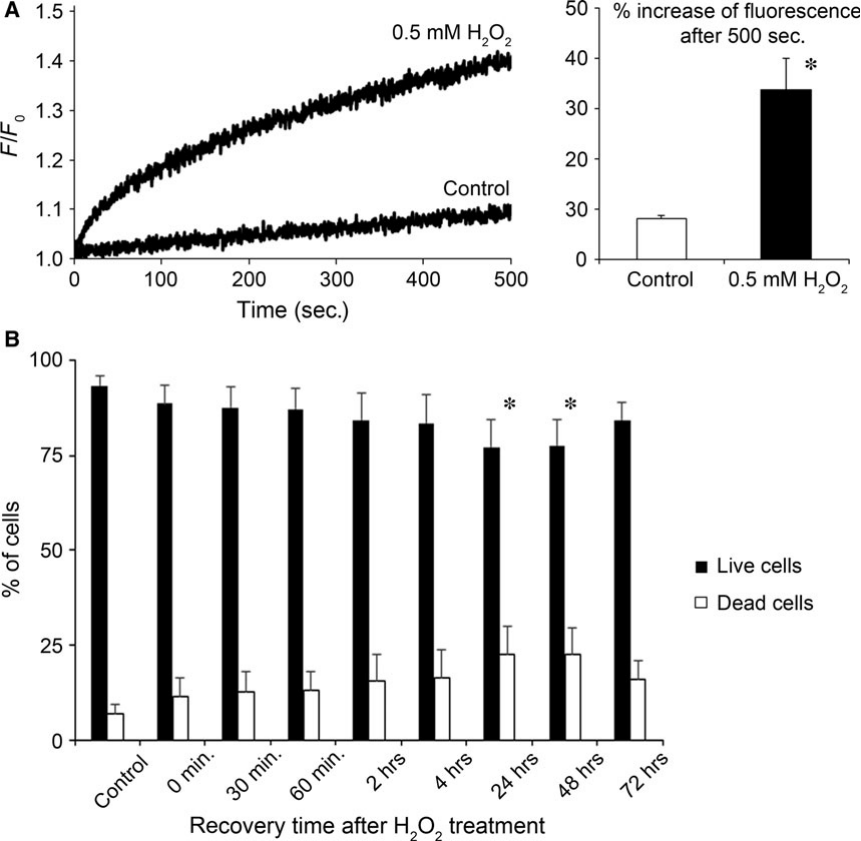
Effect of moderate oxidative stress on cell viability. (**A**) Intracellular ROS levels measured by ROS sensitive fluorescent dye CM‐H2DCFDA. SH‐SY5Y cells were treated with either 0.5 mM H_2_O_2_ or vehicle (control) starting at 0 sec. and the change in fluorescence signal was recorded for 500 sec. Representative curves of relative fluorescence (F/F_0_) are shown on the left, whereas the average increase in fluorescence signal 500 sec. after the start of the experiment is shown on the right. Data are means ± S.E.M. from three independent experiments. *P < 0.05 *versus* control. (**B**) SH‐SY5Y cells were treated with 0.5 mM H_2_O_2_ for 30 min. in serum‐free growth medium, then the medium was replaced and cells were incubated under normal growth conditions. The indicated times refer to the recovery time after the end of H_2_O_2_ treatment. At those time points, cells were collected and cell viability assay was performed to measure the percentage of living cells (Annexin V and Propidium‐Iodide negative) and dead cells (Propidium‐Iodide positive). Data are means ± S.E.M. from eight independent experiments. *P < 0.05 *versus* control.

We have used H_2_O_2_ to expose SH‐SY5Y neuroblastoma cells to oxidative stress. Figure 1A shows that the addition of 0.5 mM H_2_O_2_ markedly increased the level of intracellular ROS as measured by the ROS sensitive fluorescent dye CM‐H2DCFDA. To simulate acute oxidative stress and subsequent recovery, we exposed the cells to 0.5 mM H_2_O_2_ for 30 min. and then carefully washed out the oxidative agent from the medium to allow the cells to recover (Fig. 1B). Intracellular ROS levels were also assessed at various timepoints (1–6 hrs) after the removal of H_2_O_2_ from the media, but no significant difference could be found between control and H_2_O_2_‐treated cells (data not shown). Next, the cells were incubated under normal growth conditions for up to 72 hrs. The damage caused by oxidative stress was fully apparent at 24 hrs after the treatment (the ratio of dead cells increased to 22.6 (±7.3) % compared to 6.7 (±2.5) % of the control). The ratio of dead cells did not increase any further (22.3 (±7.1) % and 15.8 (±5.0) % after 48 and 72 hrs, respectively).

### Immunofluorescence microscopy reveals dynamic changes in protein O‐GlcNAcylation

**Figure 2:**
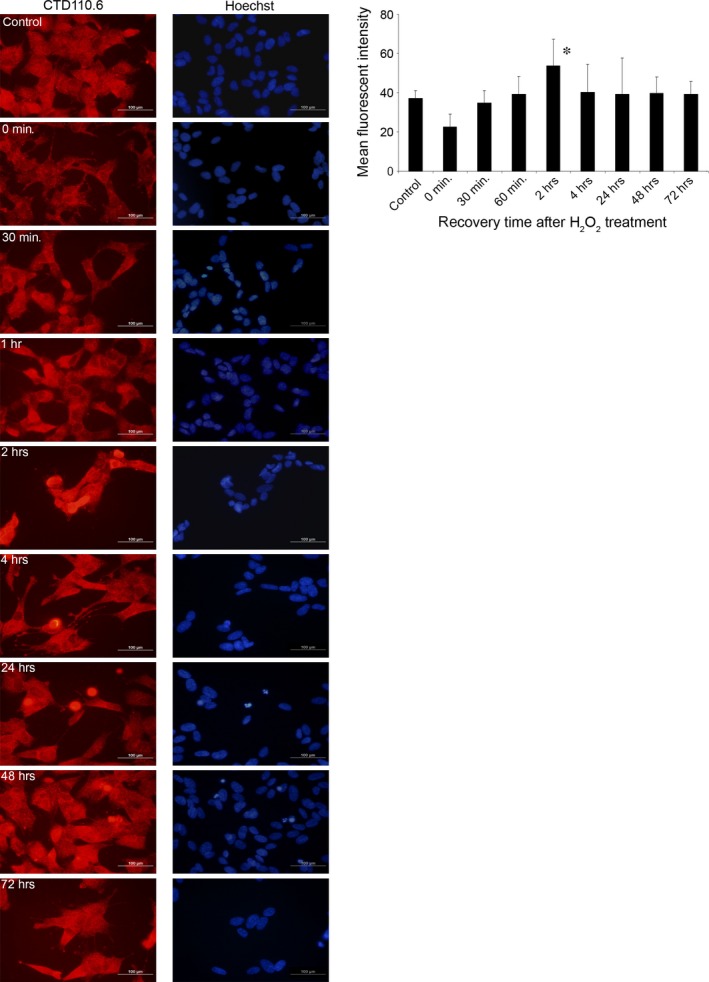
Immunofluorescence shows temporal dynamic of protein O‐GlcNAc modification after oxidative stress. Immunofluorescence labelling was performed on SH‐SY5Y cells using the anti‐O‐GlcNAc antibody CTD110.6 and Hoechst was used as a nuclear counterstain. Cells were treated with 0.5 mM H_2_O_2_ for 30 min. then replaced in growth medium to let the cells recover for up to 72 hrs. Left: representative images at the indicated times after oxidative stress are shown. Right: quantitative analysis of immunofluorescent recordings. The data are the cumulative sum from three independent experiments; average pixel intensity was collected from at least 15 cells and normalized to Hoechst counterstain. Data are shown as means ± S.E.M., *P < 0.05 *versus* 0 hr.

To assess the effect of oxidative stress induced by 30 min. treatment with 0.5 mM H_2_O_2_ on protein O‐glycosylation, SH‐SY5Y cells were formalin fixed and labelled with anti‐O‐GlcNAc monoclonal antibody (CTD110.6) after exposing them to conditions analogous to the cell survival experiments. Figure 2 shows control and stress‐exposed cells after various recovery times. Similar to other cell types, SH‐SY5Y cells showed abundant, diffuse granular cytoplasmic CTD110.6 positivity. Whereas each cell seemed to increase its overall O‐GlcNAc levels upon stress, we found some very intensely labelled cells that also appeared to have more compact and round‐shaped morphology. Based on Hoechst nuclear counterstaining, these cells were either mitotic cells or apoptotic cells, the latter more frequently appearing in the late post‐stress phase (data not shown). Fluorescence intensity was averaged for all experimental conditions and is summarized in the bar graph shown at the right of Figure 2. Although no significant increase in intensity was observed at 2 hrs compared with control, we found significant changes within stress‐exposed cells as recovery time progressed.

### Elevation in O‐GlcNAc protein and decrease/reduce in protein phosphorylation

**Figure 3:**
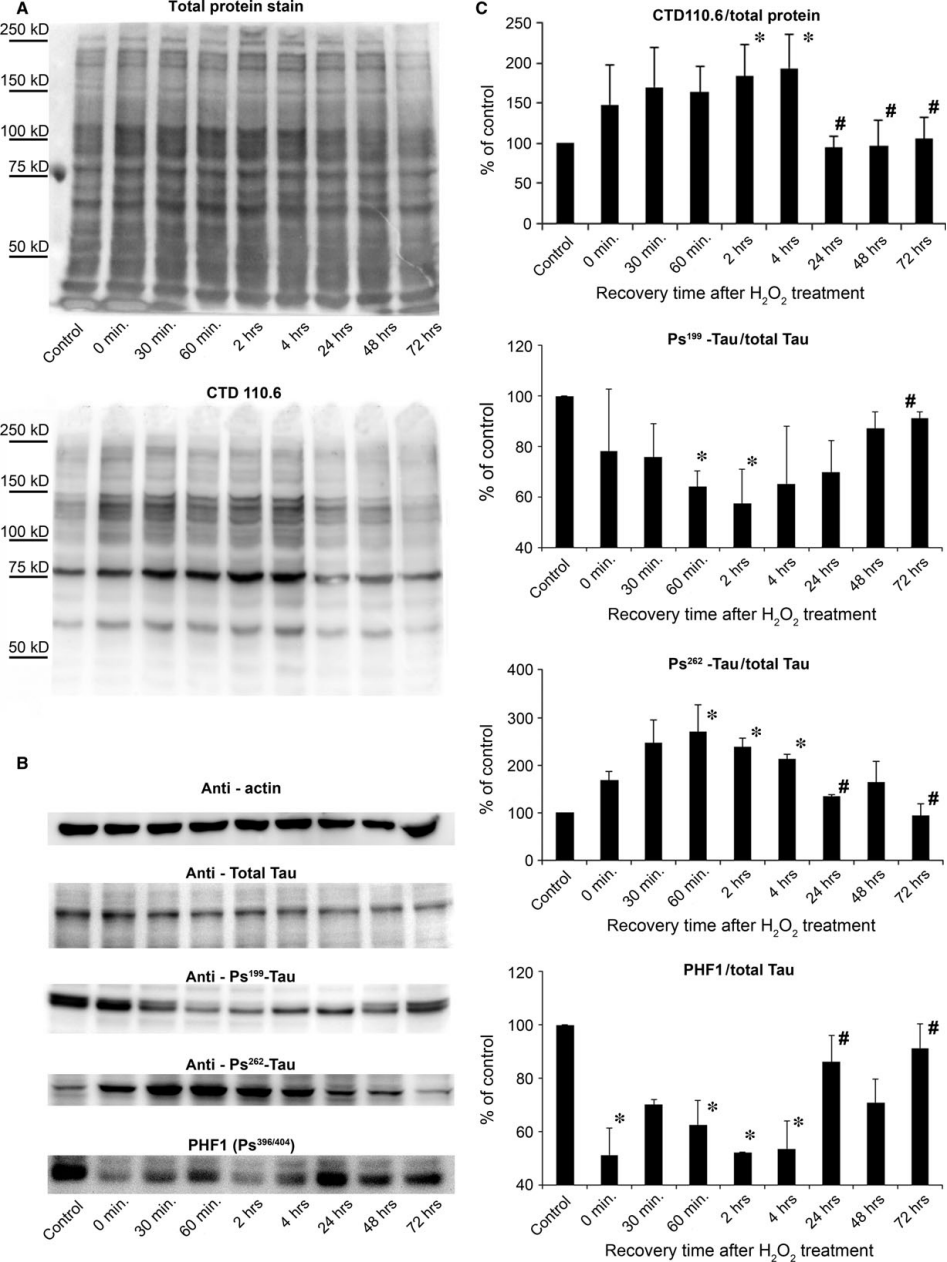
Temporal dynamics of O‐GlcNAc modification and tau phosphorylation after oxidative stress revealed by Western blot. (**A**) Western blot analysis using SYPRO Ruby Blot Total Protein Staining and CTD110.6 antibody and (**B**) actin, total tau, [Ps^199^], [Ps^262^] and PHF1 staining shows representative samples of protein extracts from SH‐SY5Y cells previously incubated for 30 min. with 0.5 mM H_2_O_2_ then replaced in normal growth medium to recover. (**C**) Densitometric analysis of the CTD110.6 and [Ps^199^], [Ps^262^] and PHF1 staining over time. Levels are expressed as a percentage of the baseline ratio. Each data point represents the average of at least three separate experiments. Data are shown as means ± S.E.M., *P < 0.05 *versus* Control, ^#^ *P* < 0.05 *versus* 2 hrs.

The dynamic changes in O‐GlcNAc levels demonstrated by immunofluorescence were confirmed by immunoblots. We found that SH‐SY5Y cells required about 2–4 hrs to reach peak level of O‐GlcNAc following oxidative stress induced by 30 min. treatment with 0.5 mM H_2_O_2_. On the long run, O‐GlcNAc levels returned to normal intensities after about 24–48 hrs (Fig. 3).

Many studies have shown an interaction between O‐GlcNAc and other signalling mechanisms, the most documented one is the reciprocal relationship between O‐GlcNAc and phosphorylation, particularly on tau proteins which play a key role in the development of Alzheimer's disease 22. As of today, scientific consensus agrees that proper O‐GlcNAcylation can prevent the hyperphosphorylation of tau proteins and subsequent formation of neurofibrillary tangles 23. In this study, we analysed the level of tau phosphorylation (Fig. 3B) using an antibody against total tau and phophospecific antibody against tau [Ps^199^], [Ps^262^] and PHF1. We found that the ratio of tau [Ps^199^] and PHF1 compared with the amount of total tau protein level significantly decreased after oxidative stress induced by 30 min. treatment with 0.5 mM H_2_O_2_. After 24–72 hrs of recovery times, both [Ps^199^] tau and PHF1 phosphorylation returned to baseline levels, which dynamic seems to be the opposite of the overall O‐GlcNAc levels’ dynamic. On the other hand, [Ps^262^] phosphorylation seemed to follow a parallel dynamic to O‐GlcNAcylation; short‐term elevation after oxidative stress followed by decreased level of phosphorylation during the recovery phase.

### Real‐time PCR analysis of GFAT and OGT mRNA expression in SH‐SY5Y neuronal cell culture

**Figure 4:**
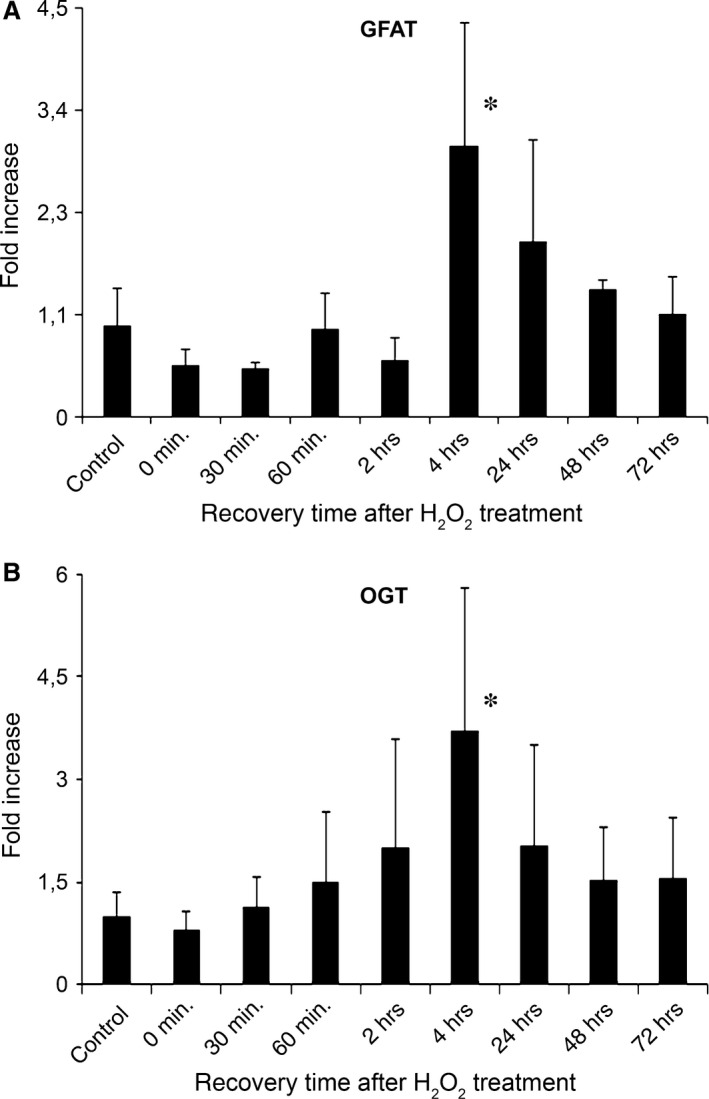
Temporal dynamics of OGT and GFAT expression after oxidative stress follow similar pattern as protein O‐GlcNAc modification. SH‐SY5Y cells were treated with 0.5 mM H_2_O_2_ for 30 min. in serum‐free growth medium, then the medium was replaced and cells were incubated under normal growth conditions until harvesting the cells. Each bar represents fold increase in relative levels of OGT or GFAT mRNA compared with control samples. **(A)** Relative levels of GFAT mRNA in cells exposed to oxidative stress at the indicated post‐stress times. **(B)** Relative levels of OGT mRNA in cells exposed to oxidative stress at the indicated post‐stress times. Data are means ± S.E.M. from at least three independent experiments. *P < 0.05 *versus* control cells.

Protein O‐GlcNAc modification is regulated by OGT which is the only enzyme responsible to the addition of the *N*‐acetyl‐glucosamine moiety to Ser/Thr residues. However, metabolic flux through the hexosamine biosynthesis pathway (HBP) also influences O‐GlcNAc levels by the regulation of the availability of UDP‐GlcNAc. The first, rate‐limiting step of HBP is catalysed by GFAT. Therefore, we analysed the mRNA expressional levels of two key enzymes related to O‐GlcNAc; GFAT (Fig. 4A) and OGT (Fig. 4B).

SH‐SY5Y cells were exposed to the same oxidative stress condition and recovery periods as described above. We found that the mRNA level of both GFAT and OGT showed a biphasic dynamic. First, GFAT and OGT mRNA was increased by up to ~3× the normal expressional rate peaking at 4 hrs post‐stress, than the expressional levels gradually decreased close to the normal levels in the next 3 days.

## Discussion

In this study we demonstrated the effect of 30 min. treatment with 0.5 mM H_2_O_2_ on the regulation of the O‐GlcNAc dynamics in SH‐SY5Y neuroblastoma cells. We have found that following this oxidative stress, protein O‐GlcNAc modification and mRNA expressional levels of GFAT and OGT, the rate‐limiting enzymes of HBP and O‐GlcNAc, respectively, are all changing in a very similar manner over time (Figs 3 and 4). O‐GlcNAc levels were increased significantly reaching peak values 2–4 hrs post‐stress. At a longer time scale – after about 24–72 hrs – the cells apparently recovered from the stress and O‐GlcNAc returned to normal levels similar to what was observed prior to stress. Interestingly, we found that tau phosphorylation changed in a reciprocal way; it decreased at 2–4 hrs after stress, but increased again after about 72 hrs following stress. These results suggest that O‐GlcNAc modification is a sensitive, dynamic marker of cellular stress and that its interaction with other intracellular processes (such as the Alzheimer's hallmark tau phosphorylation) can have both acute and chronic consequences.

O‐GlcNAc seems to regulate (or at the least influence) many intracellular processes such as cell‐cycle 24, epigenetics 25, stress adaptation 12, Ca^2+^ signalling 26 or phosphorylation 27, consequently its imbalance has been investigated in the development of many diseases. Since its discovery 28, O‐GlcNAc has been implicated either as a beneficial (*e.g*. in Alzheimer's disease 29 and ischemia reperfusion 30), or as an adverse factor (insulin resistance, diabetes 22 and malignant disorders 31). Chronic diseases in which O‐GlcNAc seems to be involved take years or even decades to develop; however, O‐GlcNAc also has relevance in cellular mechanisms that take place at a much shorter time scale. In particular, stress response and the role of O‐GlcNAc in cellular adaptation to various stresses such as hypoxia or oxidative stress has been intensively studied 32, 33. Most of these studies were conducted on cardiomyocytes and on isolated perfused hearts. Overwhelming number of evidence from these studies suggests two main conclusions; (*i*) overall O‐GlcNAc level tends to increase shortly after stress, (*ii*) increasing the level of O‐GlcNAc seems to protect from the deleterious effect of stress 30, 34, 35, 36. Theories have been put forward to explain the protective effect of O‐GlcNAc, such as HSP activation or modulation of Ca^2+^ signalling 32, 37, but the complex pro‐survival mechanism of O‐GlcNAc remains to be elucidated. In contrast to cardiac tissue, relatively little is known about O‐GlcNAc's behaviour during stress in the neuronal tissue. Interestingly, the available literature suggests that O‐GlcNAc is rather down‐regulated by hypotonic 38, or LPS‐induced stress 39. On the other hand (and paradoxically), Cheung and Hart 40 found that glucose deprivation significantly increased O‐GlcNAc levels in Neuro‐2a murine neuroblastoma cells. In our present experiments we have found that in SH‐SY5Y cell line, which is a human neuroblastoma cell line, O‐GlcNAc levels behave in a similar manner as in cardiomyocytes (Fig. 3). Moreover, O‐GlcNAc elevation was accompanied by the up‐regulation of key regulators of the HBP pathway, to support the cells’ need for more O‐GlcNAc (Fig. 4). Thus, our data demonstrate that O‐GlcNAc increases upon oxidative stress are an actively regulated process in neuronal cells.

Contrary to the relatively unexplored behaviour of O‐GlcNAc during acute stress response in neuronal cells, its reciprocal relationship with phosphorylation and imbalance in Alzheimer's disease is well‐established 19, 22. According to the generally accepted view, the dynamic balance between phosphorylation and O‐GlcNAc modification on tau proteins in Alzheimer's patients is shifted in favour of the phosphorylation which will result in hyperphosphorylated tau and neurofibrillary tangle deposition. Stress, and especially oxidative stress is also strongly implicated in the pathophysiology of Alzheimer's disease, however, the exact relationship between oxidative stress and tau phosphorylation is controversial. Some of the oxidative agents cause hyperphosphorylation, whereas others induce de‐phosphorylation of tau 41. Multiple studies also indicated that tau hyperphosphorylation can follow, but also precede oxidative stress 42. To our knowledge, O‐GlcNAc modification of tau has not been investigated in oxidative stress before, however, common sense dictates that the reciprocal relationship between phosphorylation and O‐GlcNAc should be maintained also under this condition. In our experiments, we have found that 30 min. long oxidative stress by H_2_O_2_ resulted in temporal decrease of tau phosphorylation at site S199 and PHF1, but reciprocal elevation in O‐GlcNAc modified proteins (Fig. 3). Interestingly, the phosphorylation at site S262 showed similar dynamic pattern as O‐GlcNAc levels. Although the interplay between O‐GlcNAc and phosphorylation is usually characterized by same site competition, other mechanism such as proximal site competition or proximal site occupation is also known 13. Increased phosphorylation at S262 (and possibly at other sites as well) after oxidative stress despite elevated level of O‐GlcNAc could be the result that either O‐GlcNAc is not competing or even contributing to phosphorylation under various conditions.

Although our O‐GlcNAc data collected from SH‐SY5Y neuroblastoma exposed to H_2_O_2_ induced oxidative stress are in accordance with the data found in cardiomyocytes and also demonstrate the reciprocal relationship between O‐GlcNAc and specific tau phosphorylation sites, the question whether these effects observed at short term could be understood and interpreted also under chronic conditions remains to be answered. In Alzheimer's disease, despite oxidative stress is a major factor in the pathophysiology, the dynamic of O‐GlcNAc seems to be reversed. It is also important to note that diabetes is a risk factor for Alzheimer's disease 43. One of the mechanism by which diabetes could accelerate the development of AD is increased ROS production. Insulin resistance in type 2 diabetes was also found to be associated with AD. How big of a role O‐GlcNAc plays in these processes and how these processes are related to the regulation of tau phosphorylation still needs to be addressed. Nevertheless, we think that studying O‐GlcNAc modification simultaneously with tau phoshorylation in oxidative stress‐exposed neuronal cells should significantly contribute to the understanding of the development of Alzheimer's disease.

A number of studies deal with the role of O‐GlcNAc in various stress situations 12, 13, 32, 33. Here, we demonstrated, for the first time, that treatment with 0.5 mM H_2_O_2_ for 30 min. to induce oxidative stress causes a rapid, temporary increase in O‐GlcNAc in SH‐SY5Y neuroblastoma cells. We have also shown that the dynamic changes in protein O‐GlcNAc modification can behave in an inverse relationship with tau phosphorylation during recovery from oxidative stress. Taken together, we think that O‐GlcNAc regulation is an important mechanism in stress adaptation and it is conceivable that protein O‐GlcNAc modification is part of a complex system that influences not only short‐term effects but also chronic pathophysiologic events such as Alzheimer's disease.

## Conflict of interest

The authors confirm that there are no conflicts of interest.
